# TechBio 3.0 closes the drug discovery loop with multimodal AI and generative chemistry

**DOI:** 10.1038/s44386-026-00078-x

**Published:** 2026-10-09

**Authors:** Krish Ramadurai, Abhirup Banerjee

**Affiliations:** Institute of Biomedical Engineering, Department of Engineering Science, University of Oxford, Oxford, UK

**Keywords:** Computational biology and bioinformatics, Drug discovery

## Abstract

Artificial intelligence (AI) has transformed molecular prediction and design, yet these advances remain only partially integrated with experimental discovery. We define TechBio 3.0 as an emerging paradigm for closed-loop drug discovery that connects multimodal molecular representations, generative chemistry, and automated experimentation. We examine its early preclinical and clinical evidence, identify remaining technical and translational barriers, and argue that this convergence is beginning to define biotechnology’s next era.

## Introduction

### The pharmaceutical productivity crisis

Over the past five decades, pharmaceutical research and development spending has increased substantially while approval rates have remained largely stagnant^1,2^. The average cost of bringing a new drug to market now exceeds $2 billion, and development timelines extend beyond 12 years from initial target identification to regulatory approval^3^. Notably, almost 90% of drug candidates entering clinical trials fail to achieve approval, with safety concerns accounting for a substantial portion of these failures across all stages, from preclinical testing through post-marketing surveillance^4,5^. Late-stage failures are especially costly, given the scale of investment in clinical trials, manufacturing, and regulatory preparation before toxicity signals emerge. This inefficiency has heightened interest in computational strategies to identify promising candidates earlier in the pipeline, enabling the exclusion of potentially toxic compounds before costly clinical testing.

One of the main reasons for the productivity crisis in drug discovery is the challenge of predicting how drug molecules will behave in complex biological systems. A single compound might bind to its intended target, but it can also interact with many off-target proteins, build up in certain tissues, be metabolized into reactive intermediates, and trigger stress responses in vulnerable cell types^6,7^. Capturing this wide range of biological effects from molecular structure alone requires computational methods that can represent multiple facets of molecular behavior. Traditional quantitative structure-activity relationship (SAR) models simplify three-dimensional (3D) structures into fixed-length descriptor vectors. While this approach can work well for simple endpoints, it often falls short of capturing the spatial arrangement and conformational changes that are important for safety^8,9^.

### The evolution of biotech to TechBio

Technology-enabled biotechnology, or “TechBio,” has evolved over two decades as computational methods have become increasingly embedded in pharmaceutical research^10^. The early 2000s saw breakthroughs in high-throughput screening and combinatorial chemistry, which enabled the rapid generation and evaluation of large compound libraries. Although these innovations transformed the lead identification process, they did not translate into higher approval rates^11^. Many compounds identified through screening ultimately failed because of pharmacokinetic liabilities, off-target effects, or mechanism-based toxicities that screening assays could not predict. Although bioinformatics tools have become increasingly embedded in research workflows, drug discovery remained largely sequential and experimentally driven.

TechBio 1.0 (prediction) took shape between 2015 and 2019, fueled by machine learning to predict molecular properties^12,13^. Deep learning models trained on chemical structure data yielded better predictions for binding affinity, solubility, metabolic stability, and early toxicity endpoints^14,15^. These systems primarily served as tools for ranking or filtering compounds within traditional drug discovery pipelines. While they improved decision-making during preclinical optimization, their influence on clinical outcomes was limited by data availability and the lack of mature integration with experimental work. This phase helped establish artificial intelligence (AI) as a valuable tool for drug discovery, but its role remained largely predictive and did not fundamentally change how the process was functionally structured.

TechBio 2.0 (acceleration) emerged between 2020 and 2023, as generative models and end-to-end AI platforms accelerated the discovery process. AI was now being used earlier in the pipeline, including for target identification and the design of brand-new molecules. Several programs reported faster progress from target nomination to lead candidate selection. For instance, Insilico Medicine advanced an idiopathic pulmonary fibrosis (IPF) program from target identification to preclinical candidate nomination in about 18 months, whereas this process traditionally takes 3–5 years^16^. The clinical development of Rentosertib, a Traf2- and Nck-interacting kinase (TNIK) inhibitor designed by generative AI, stands out as one of the first examples of an AI-enabled drug advancing into clinical trials^17^. Despite these advances, workflows in this phase were still only partially integrated, with predictive, generative, and experimental components working together but not continuously optimized within a unified feedback system.

**Fig. 1:**
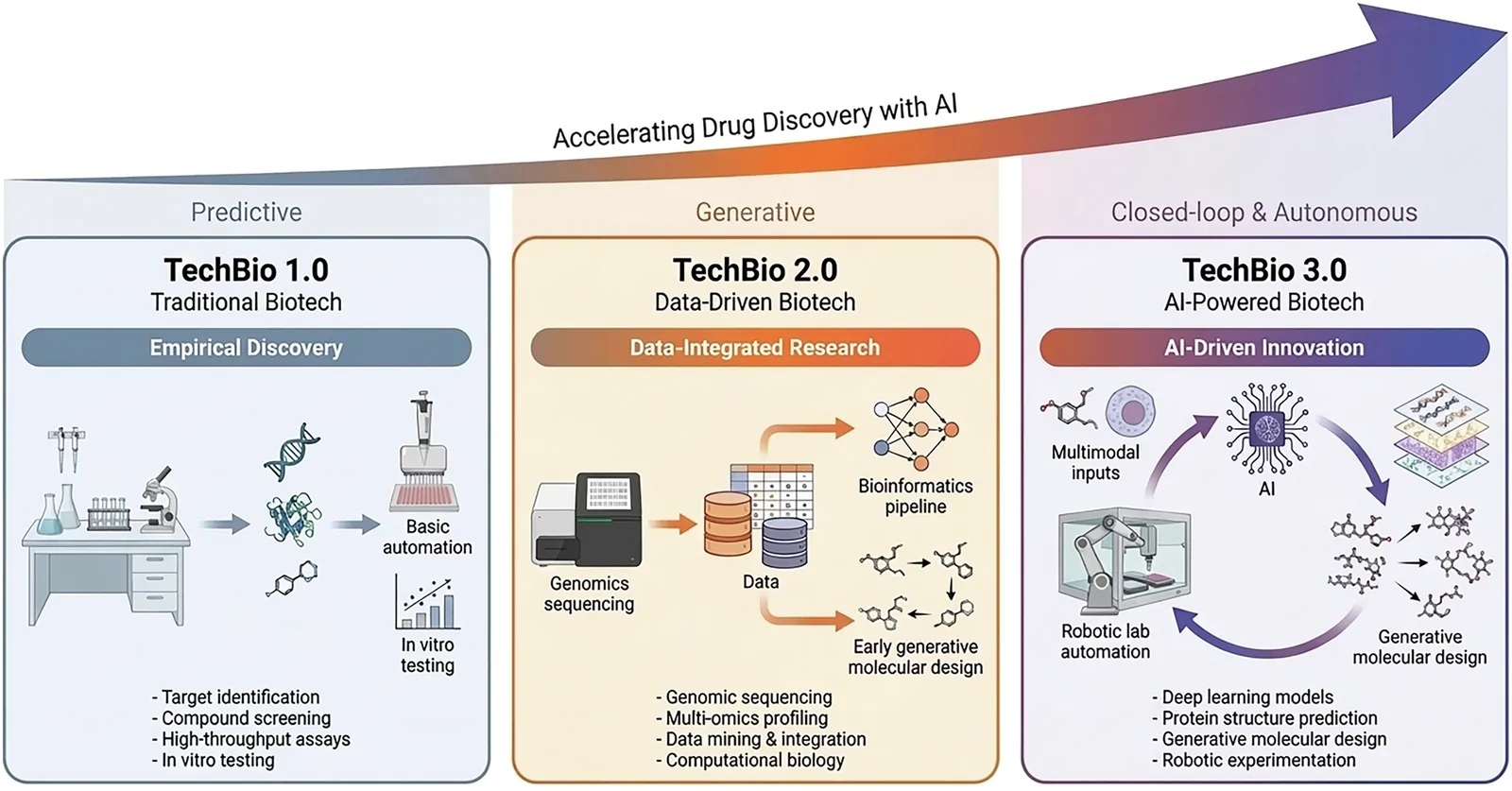
Evolution of AI-driven drug discovery across three generations, from TechBio 1.0 to TechBio 3.0. The schematic illustrates how recent generations of artificial intelligence (AI) drug discovery have evolved from predictive AI models that estimated properties (TechBio 1.0), to accelerating discovery workflows through generative-design molecule creation and target discovery (TechBio 2.0), toward closing the loop with multimodal and laboratory-integrated discovery platforms that couple prediction and generation with experimental feedback (TechBio 3.0). The large arrow represents the gradual adoption of AI into the drug-discovery workflow. Icons within each panel highlight the representative capabilities of that generation: simple automation and in vitro assays for TechBio 1.0, bioinformatics pipelines and genomic sequencing for TechBio 2.0, and multimodal data inputs, generative molecular design, and robotic lab automation for TechBio 3.0.

We define “TechBio 3.0” (closed-loop generative multimodal integration) as a structural transition emerging in the current phase of AI-native drug discovery. This new era is characterized by the merging of multimodal molecular representations, generative design systems, and laboratory-integrated feedback frameworks. AI-native platforms now bring together chemical structures, protein interaction modeling, transcriptomic response prediction, and perturbation data into unified embeddings that allow for context-dependent predictions of efficacy and safety. Generative models use these multimodal outputs directly, building toxicity and pharmacokinetic constraints into the design process itself, rather than filtering out candidates after the fact. Just as importantly, high-throughput experimental platforms and lab automation systems feed structured data back into the models, driving refinement through active learning and uncertainty-aware optimization. In contrast to earlier stages, TechBio 3.0 views drug discovery as an adaptive control system, rather than a linear process. Historically, one of the biggest challenges for TechBio has been bridging the gap between computer-based predictions and actual biological or clinical outcomes. By integrating multimodal AI models with experimental perturbation datasets and human biological data, TechBio 3.0 takes an engineering-driven approach to improve translatability in a field where over 90% of drug candidates still fail to reach approval^4^ (Fig. 1). Unlike TechBio 2.0, where predictive, generative, and experimental components remain loosely coupled, TechBio 3.0 is defined by tight integration of algorithms and experiments, in which model outputs directly inform synthesis and assay design, and experimental data are continuously incorporated to update model parameters and uncertainty estimates.

### Scope and structure of this review

This review examines the technological innovations that define TechBio 3.0, focusing on two converging methodologies that, together, enable more comprehensive and actionable predictions. Multimodal AI systems integrate visual representations, graph-based topologies, mechanistic simulations, and predicted transcriptomic responses to capture complementary aspects of molecular behavior that no single approach can fully capture^18,19^. Generative chemistry platforms employ AI to do more than just predict the properties of existing compounds; they also design entirely new molecular structures optimized for therapeutic effect and safety^20,21^. By combining these methodologies, a truly integrated workflow is established, moving from toxicity prediction through to molecular optimization. This allows pharmaceutical scientists to proactively identify compounds with undesirable profiles and create safer alternatives for experimental testing.

This review is organized into seven sections following the introduction. We first explore the biological and technical reasons behind the move toward multimodal approaches for molecular representation. We then cover the latest architectural innovations in multimodal neural networks for drug discovery, including attention-based fusion methods that offer compound-specific interpretability, a crucial requirement for regulatory approval. Next, we examine generative chemistry strategies, ranging from traditional rule-based bioisosteric transformations to modern, data-driven neural generative models. We discuss how prediction and generation are being brought together into unified, closed-loop optimization workflows. We highlight clinical and commercial validation of TechBio 3.0 through selected case studies. Finally, we address ongoing challenges spanning data access, evolving regulatory frameworks, and gaps in experimental validation that need to be overcome to unlock the full promise of end-to-end, AI-driven drug discovery.

## Foundations of multimodal molecular representation

### The biological complexity underlying drug toxicity

Understanding why multimodal approaches are necessary begins with recognizing the complexity of drug interactions with living systems. A pharmaceutical compound may bind to its intended target through specific 3D interactions, such as hydrogen bonds, hydrophobic contacts, and electrostatic complementarity^22^. Yet, the same compound might also inhibit off-target enzymes through distinct structural features, disrupt cell membranes due to its physicochemical properties, trigger stress responses if it is metabolized into reactive intermediates, or accumulate in certain tissues via transporter-mediated uptake^23,24^. Capturing this full range of effects requires models that integrate structural, physicochemical, and biological information, rather than focusing on just one type of data. Hepatotoxicity is a clear example of this complexity and remains a major reason why drugs fail or are withdrawn from the market^25^. Liver injury can occur through at least four different mechanisms: (i) formation of reactive metabolites, which depends on the arrangement of functional groups and their accessibility to cytochrome P450 enzymes; (ii) mitochondrial dysfunction, which is influenced by the compound’s physicochemical properties that affect its accumulation within mitochondria, the site of oxidative phosphorylation; (iii) inhibition of bile salt export pumps, which relies on the molecule’s shape and electrostatic features that facilitate transporter binding; and (iv) immune-mediated injury, which results from the formation of haptens when the drug covalently binds to proteins, prompting the immune system to see the modified protein as foreign^26–29^. Since no single molecular representation can capture all these factors, multimodal approaches are often more effective than models that rely on just one type of information.

To clarify the connection between biological mechanisms and model inputs, Tables 1, 2 and 3 map each hepatotoxicity mechanism discussed above to the molecular or biological modality most likely to capture it. Hepatotoxicity, or drug-induced liver injury (DILI), is a strong example of why multimodal models are needed. DILI can arise from structural features of a molecule, such as reactive groups that drive toxic metabolite formation, but it can also emerge from downstream biological responses, including stress-pathway activation and immune-mediated injury^22,25^. A single data type is unlikely to capture all of this biology. Consistent with this, a model combining structural fingerprints with learned Simplified Molecular Input Line Entry System (SMILES) sequence representations outperformed either modality alone in DILI prediction, and an ablation analysis showed that the two inputs provided complementary information^30^. Similarly, deep learning models trained on high-throughput L1000 transcriptomic profiles predicted DILI more accurately than classical descriptor-based approaches^31^. We therefore retain hepatotoxicity as a dedicated case study because it provides one of the clearest empirical arguments for multimodal integration. At the same time, the same logic extends beyond DILI to the broader Absorption, Distribution, Metabolism, Excretion, and Toxicity (ADMET), efficacy, and selectivity landscape; the cardiotoxicity comparison in Table 4 provides one example.

**Table 1:** Mapping of hepatotoxicity mechanisms to the molecular modalities and encoders expected to capture them, with mechanistic rationale

Hepatotoxicity mechanism	Molecular/Biological driver	Best-suited modality (encoder)	Rationale
(i) Reactive metabolite formation^22,28^	Functional-group arrangement; accessibility to CYP450	2D depiction (CNN) + molecular graph (GNN)^29,45^	Molecular structure allows capture of experimentally observed toxicity “red flags” (e.g., structural alerts/toxicophores/reactive groups) while graph message passing allows for modeling of electronic effects that affect bioactivation
(ii) Mitochondrial dysfunction^23^	Lipophilicity, charge, accumulation in mitochondria	Mechanistic/physicochemical encoder (ODE)^33^	Mitochondrial dynamics features model toxicity manifestation dynamics (ATP depletion, ROS production, and membrane potential dissipation) rather than assuming toxicity as an inherent chemical property
(iii) BSEP (bile-salt export pump) inhibition^24^	Molecular shape + electrostatic complementarity for transporter binding	Molecular graph/3D + physicochemical descriptors^32,48^	Graph topology and physicochemical features model sterics, electrostatics, and accumulation features that impact transporter binding and resulting intracellular exposures
(iv) Immune-mediated (idiosyncratic) injury^25^	Covalent hapten formation; downstream immune activation	Transcriptomic signature + reactive-group graph features^35,36^	Transcriptomic signatures model the downstream biological consequences, such as stress and immune responses

**Table 2:** Complementary molecular representations, their encoders, and the toxicity-relevant information each contributes

Representation	Encoder	Information captured	Toxicity relevance	Key limitation
2D structure image^45,46^	CNN (e.g., ResNet-18)	Visual motifs, toxicophore patterns	Quinones, aromatic amines, structural alerts^22,28^	Sensitive to how the molecule is drawn or oriented, and provides limited 3D structural information
Molecular graph^29,32^	GNN/GAT (graph attention; message passing)	Connectivity, electronic propagation	Reactivity, resonance/inductive effects	Captures chemical connectivity well, but may miss long-range interactions and 3D conformational effects
Physicochemical/mechanistic^33,48^	Descriptor + ODE network	Dynamic biological trajectories (ATP, ROS)	Mitochondrial and time-dependent toxicity^23^	Depends on curated biological assumptions and kinetic parameters, and usually simplifies complex biology
Transcriptomic signature^35,36^	Structure-to-expression encoder	Pathway-level cellular response	Immune-mediated and stress-pathway injury^25^	Sparse experimental coverage; cell-line dependence

**Table 3:** Summary of multimodal neural architectures: input modality, representative architecture, captured information, interpretability mechanism, strengths and limitations

Modality (input)	Representative architecture	Information captured	Interpretability	Strengths	Limitations
Molecular image (2D)^45,46^	CNN/ResNet-18 + spatial attention	Visual toxicophores	Grad-CAM heatmaps^60^	Transfer learning; intuitive maps	Depiction-dependent; weak 3D
Molecular graph^29,32^	GNN/GAT (3 MP layers)	Topology, electronic propagation	Edge/node attention^59^	Permutation-invariant; chemically grounded	Over-smoothing; limited long-range
Physicochemical + dynamics^33,48^	Descriptor net + Neural ODE	Time-course biological state	Trajectory attribution	Mechanistic; data-efficient	Needs kinetic priors
Transcriptomic^35,36^	Structure-to-expression MLP/encoder	Pathway activation	Gene-level attribution	Captures downstream effects	Sparse labels; cell-line bias
Quantum/physics-informed^50–53^	EGNN/equivariant transformer (qcGEM, QuADMET-Former)	Electronic structure, electrostatics	Per-atom QM attribution	OOD robustness; low label need	Compute cost; QM data curation

**Table 4:** Comparison of multimodal fusion strategies for tasks such as hepatotoxicity and cardiotoxicity (hERG) prediction, highlighting the interpretability and accuracy advantages of hierarchical attention fusion

Fusion strategy	Mechanism	Strengths	Weaknesses	Best-suited scenario	Interpretability
Early (feature-level)^54^	Concatenate raw features pre-model^109^	Fast; captures low-level cross-modal correlations	Ignores modality interactions; sensitive to scale/missingness	Aligned, complete modalities	Low
Late (decision-level)^54^	Combine per-modality outputs (avg/vote)	Robust to missing modalities; modular	Misses synergistic joint effects; static weights	Heterogeneous or partially missing data	Moderate (per-modality)
Hierarchical attention^55^	Dynamic, compound-specific modality + feature weights	Captures synergy; adaptive; highest accuracy reported	More parameters/data; harder to train	Mechanism-dependent toxicity (e.g., DILI vs hERG)^19^	High (modality + feature level)^59^

### Complementary information encoded by different molecular representations

**Fig. 2:**
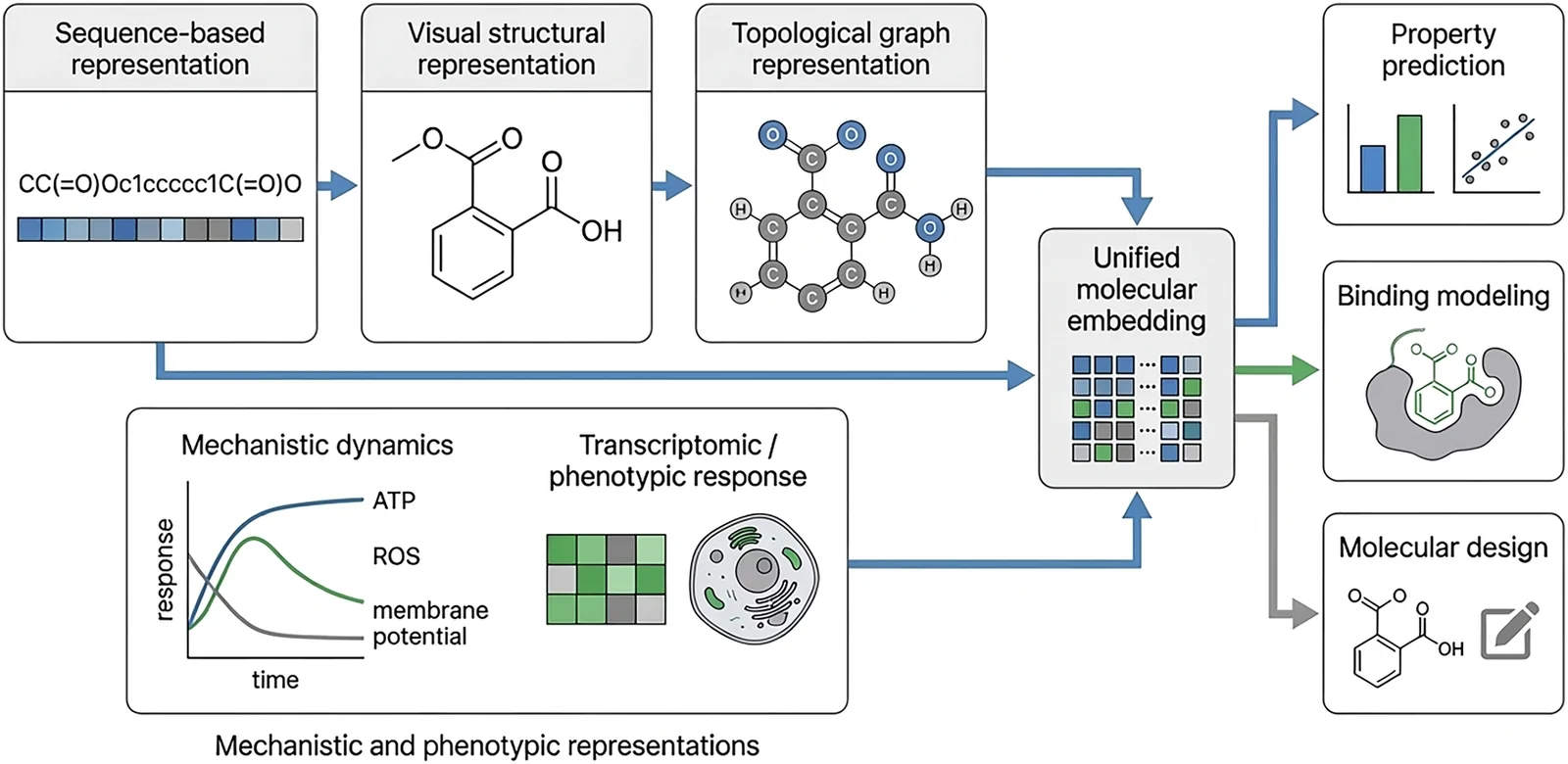
Complementary molecular representations in multimodal AI-driven drug discovery. Schematic illustrating how diverse molecular and biological representations capture complementary aspects of drug behavior and are integrated into unified molecular embedding. Sequence-based (SMILES), visual structural, and topological graph representations encode molecular identity and connectivity, while mechanistic and phenotypic representations provide dynamic cellular and transcriptomic context. Multimodal AI models integrate these inputs into a shared embedding space that links molecular structure with biological effects. This unified representation enables downstream tasks, including property prediction, binding modeling, and molecular design. The framework decouples molecular structure and biological activity. Structural inputs SMILES strings, 2D images, and molecular graphs are shown in the top rows. Mechanistic/phenotypic data are shown in the bottom rows. Incoming blue arrows converge into multimodal fusion, where the different views are combined into a joint molecular embedding.

Each type of molecular representation captures unique information, and these perspectives complement one another rather than simply repeating the same details^32^ (Fig. 2). Standardized 2D molecular depictions depict the arrangement of atoms and functional groups in ways familiar to medicinal chemists^33^. When Convolutional Neural Networks (CNNs) analyze these images, they can detect visual patterns associated with toxicity, such as quinone-like structures where ketone groups are positioned ortho- or para- to each other on aromatic rings, or aromatic amines that can be metabolically activated^34^. These visual formats help capture structural motifs that medicinal chemists often use as heuristics. In molecular graphs, atoms are represented as nodes and bonds as edges, which encodes the connectivity and identity of the molecule regardless of its drawn orientation or depiction^35^. Graph Neural Networks (GNNs) learn how properties propagate through a molecule’s topology, capturing how changes in one part of the molecule can influence reactivity elsewhere, even several bonds away. This includes electronic effects, such as resonance donation and inductive withdrawal, that propagate through conjugated systems and can be captured through graph-based message passing^36^. Attention mechanisms within these networks can highlight which atoms and bonds have the biggest impact on predictions, making the results more interpretable by linking them to meaningful chemical substructures.

Mechanistic simulations using Ordinary Differential Equations (ODEs) model how biological systems dynamically respond to compound exposure over time^37^. By simulating processes spanning Adenosine Triphosphate (ATP) production and consumption, Reactive Oxygen Species (ROS) generation during oxidative stress, changes in membrane potential due to disrupted proton gradients, and the activation of cell death pathways, these models can capture toxicity arising from dynamic changes rather than static molecular features^38^. In many cases, toxicity is not caused by a single structural motif, but emerges from cumulative effects observed over simulated exposure timelines. Transcriptomic signatures, whether measured experimentally or predicted from structure, capture how cells respond to compounds at the level of gene regulation^39,40^. Shifts in the expression of key toxicity-related genes can reveal activation of stress-response pathways such as the unfolded protein response, Nuclear Factor Erythroid 2-like 2 (NRF2)-mediated antioxidant defense, or the p53-mediated DNA damage response^41^. By focusing on downstream biological effects rather than structure alone, transcriptomic representations can identify compounds that elicit similar cellular responses, even when their chemical structures differ markedly. These approaches are supported by large-scale chemical transcriptomics platforms and curated drug libraries, which enable systematic mapping of compounds to cellular responses^42,43^.

## Multimodal neural architectures for drug discovery

### Specialized encoder architectures for each molecular representation

The foundations discussed above motivate the use of complementary molecular and biological representations for toxicity prediction. In this section, we dive into how we encode those representations as learnable embeddings that can then be fused into a joint prediction space. Each modality-specific encoder transforms diverse inputs into fixed-size embeddings that retain toxicity-relevant information while remaining compatible with downstream data fusion^44^. Architectural decisions are tailored to the data structure and to the inductive biases best suited to each type of representation. For molecular images, CNN encoders often employ transfer learning from ImageNet, initializing weights with parameters pretrained on natural images before fine-tuning on chemical depictions^45,46^. This strategy leverages broad visual feature extraction while adapting to the unique aspects of chemical structure. The ResNet architecture, which uses skip connections to stabilize deep training, has proven effective. ResNet-18 achieves a good balance between model capacity and training efficiency for typical pharmaceutical datasets^45^. Spatial attention mechanisms further enable the network to focus on specific molecular regions relevant to toxicity, generating interpretable attention maps that highlight predicted toxicophores.

GNN encoders use message-passing architectures in which node representations are iteratively updated by aggregating information from neighboring atoms^26,29^. Graph Attention Networks (GATs) extend this framework by introducing learned attention weights that modulate each neighbor’s relative contribution, enabling the model to prioritize chemically relevant interactions^32^. Node features typically include atomic descriptors such as element type, formal charge, hybridization state, aromaticity, degree, and ring membership, while edge features encode bond order, conjugation, and stereochemistry^32^. In practice, three message-passing layers are often sufficient to capture local substructure interactions in drug-like molecular graphs. Inductive representation learning approaches further extend graph-based modeling capabilities by enabling generalization to large, heterogeneous molecular graphs^47^.

Mechanistic encoders, which combine physics-based modeling with deep learning, begin by extracting physicochemical descriptors from molecular structures, typically generating 50 or more features such as molecular weight, partition coefficients, polar surface area, and hydrogen-bond counts^48^. A parameter-mapping network then turns these descriptors into specific values for each compound. These values are used in a set of linked equations that simulate how biological systems respond over time^33^. State variables might include ATP concentration, ROS levels, membrane potential, and apoptotic signaling. Numerical integration produces time-course trajectories of simulated exposure, which are summarized into statistics that form the mechanistic embedding. Transcriptomic encoders address the challenge posed by the unavailability of experimentally derived gene expression signatures for most screening compounds^35,36^. These encoders predict transcriptomic responses from molecular structures, learning mappings from SMILES strings to gene-expression principal components across multiple cell types, including HepG2 hepatocytes, A549 lung cells, and MCF7 breast cancer cells. Training data are drawn from resources such as the Connectivity Map and the TAHOE-100M dataset^49^, which provide matched compound-expression pairs across different doses, time points, and cell types^35–37^.

In addition to using classical molecular descriptors as inputs to ML models, another trend is directly encoding quantum-mechanical information into molecular encoders. By imbuing models with physical priors^48^, we can both reduce reliance on large labeled datasets and increase the likelihood that they will learn non-local electronic effects that may be missed by purely data-driven TechBio 2.0 methods. One example of this is quantum chemistry-aware graph models such as qcGEM^50^, which supplement message passing with electronic-structure-based features like partial charges, HOMO-LUMO gaps, and electrostatic potentials. In a similar vein, QuADMET-Former^51^, which is pretrained as an equivariant graph transformer on atom-in-a-molecule quantum properties, shows improved ADMET prediction on the Therapeutic Data Commons, Biogen ADME, and OpenADMET benchmarks. Other related works include LiTENexus^52^ and 3D molecular representations constructed from electron density^53^. These approaches hint towards how a more physically motivated flavor of physics-data fusion can help bolster TechBio 3.0 by enabling better extrapolation to chemistries not covered by the relatively sparse set of labeled ADMET data.

### Fusion mechanisms for integrating multiple modalities

The way information from different sources is combined affects both the accuracy and clarity of predictions, and researchers are actively exploring the best approaches for integrating these diverse datasets^54^. Early fusion merges information from all sources into a single set but does not account for interactions between sources. While this method is fast, it can overlook important connections, especially when certain types of information are more relevant for specific compounds^54,55^. In contrast, late fusion treats each data source separately before combining the results, often via averaging or voting, allowing each source to contribute its strengths and yielding more balanced predictions. However, late fusion may miss synergistic effects that require joint optimization, and static combination weights may not reflect differences in relevance between compounds. Hierarchical attention fusion addresses this by providing a more flexible, advanced solution that assigns dynamic, compound-specific weights^55^. The first attention layer determines the degree of influence each data type should have on the prediction for a given compound. For example, the model can emphasize mechanistic features when toxicity is likely due to metabolic activation, or prioritize structural features when toxicity is linked to known toxicophores. A second attention layer within each data type identifies the specific features that most strongly influence the prediction. This two-step approach enhances interpretability by clarifying not only which types of data were most important, but also which details within those data sources had the greatest impact on the prediction. Protein-side features derived from unified co-folding models (discussed under Emerging Technical Frontiers) can likewise be incorporated as an additional modality within this fusion layer^56,57^.

### Model interpretability for regulatory and scientific decision-making

In pharmaceutical safety assessment, it is not enough for a model to be accurate, as it must also provide a mechanistic explanation for its predictions^58^. Regulatory agencies, including the U.S. Food and Drug Administration (FDA) and the European Medicines Agency (EMA), are increasingly recognizing computational methods as valid parts of safety evaluations, but they expect scientific reasoning that links predictions to underlying mechanisms. Hierarchical attention fusion architectures meet this need by producing predictions along with attention visualizations that can be included in regulatory submissions^59^. These models offer several layers of insight: at the modality level, they show which types of molecular information contributed most to a prediction, clarifying whether toxicity was linked to structural features, mechanistic behaviors, or pathway activations. Within each modality, feature-level analysis pinpoints the specific molecular substructures, trajectory features, or pathway components that influenced the outcome. At the compound level, attention visualizations highlight the atoms, bonds, and functional groups associated with predicted toxicity. For CNN-based models, techniques such as Gradient-weighted Class Activation Mapping (Grad-CAM) generate spatial heatmaps on molecular images, highlighting the regions most important for prediction^60^. GNNs use edge and node attention mechanisms to highlight the molecular substructures most strongly linked to toxicity^32,46^. Mechanistic models use trajectory analysis to identify which simulated biological state variables are most associated with toxic effects^33^. For transcriptomic encoders, gene-level attribution methods reveal which changes in gene expression had the greatest impact on the model’s predictions^37,38^.

Regulatory use of interpretable AI is no longer only theoretical. The FDA’s January 2025 draft guidance, *Considerations for the Use of Artificial Intelligence to Support Regulatory Decision-Making for Drug and Biological Products*, introduces a risk-based credibility-assessment framework in which model explanations help justify the proposed context of use^61^. The EMA’s 2023 reflection paper on AI across the medicinal-product lifecycle makes a similar point, emphasizing transparency, traceability, and human oversight^62^. An established example is the ICH M7(R1) framework for mutagenic impurities. Under this framework, sponsors can use two complementary (Q)SAR predictions in place of certain bacterial mutagenicity, or Ames, studies: one expert rule-based system that identifies mechanistic structural alerts, such as Derek Nexus, and one statistical system, such as Sarah Nexus, supported by documented expert review^63,64^. More complex toxicities remain harder to explain. Idiosyncratic immune-mediated injury, for example, remains a major limitation because current methods can highlight influential input features but usually cannot reconstruct the full causal chain from hapten formation to T-cell activation^25^.

## Generative chemistry for therapeutic optimization

### Rule-based transformations and medicinal chemistry knowledge

Generative chemistry refers to computational methods for designing molecular structures that are optimized for specific goals^65^. Rule-based transformation techniques build on decades of medicinal chemistry experience, using systematic modifications to create a wide variety of analogs through well-established chemical substitutions. Bioisosteric replacement is a key strategy in this approach, in which functional groups are swapped to keep biological activity while changing other properties^66,67^. Classic examples include replacing carboxylic acids with tetrazoles to maintain acidity and improve metabolic stability, swapping amides with sulfonamides to alter conformational flexibility, and substituting nitro groups with cyano or trifluoromethyl groups to reduce mutagenic risk while preserving electron-withdrawing effects^68^. These strategies are rooted in empirical drug optimization campaigns and reflect the accumulated practice of medicinal chemists.

Chemists also modify functional groups to address specific toxicity concerns^22^. For example, converting primary aromatic amines into secondary amines or acetamides can lower the risk of oxidation. Making Michael acceptors less reactive by adding saturation or bulky groups, and disrupting motifs prone to oxidation, can help prevent the formation of toxic quinones. Modifying the molecular scaffold, such as altering ring structures, allows chemists to retain a molecule’s core shape while adjusting its metabolism^65^. For instance, swapping a phenyl ring for a pyridine adds heteroatoms that shift polarity and metabolism, and expanding a ring can change the molecule’s preferred shape. While rule-based methods are interpretable and easy to apply in the lab, they are limited by the boundaries of existing chemical knowledge. Molecular developability is also influenced by structural features such as aromatic ring count, which has been associated with increased risk of attrition in drug design^69^.

### Generative models for de novo molecular design

**Fig. 3:**
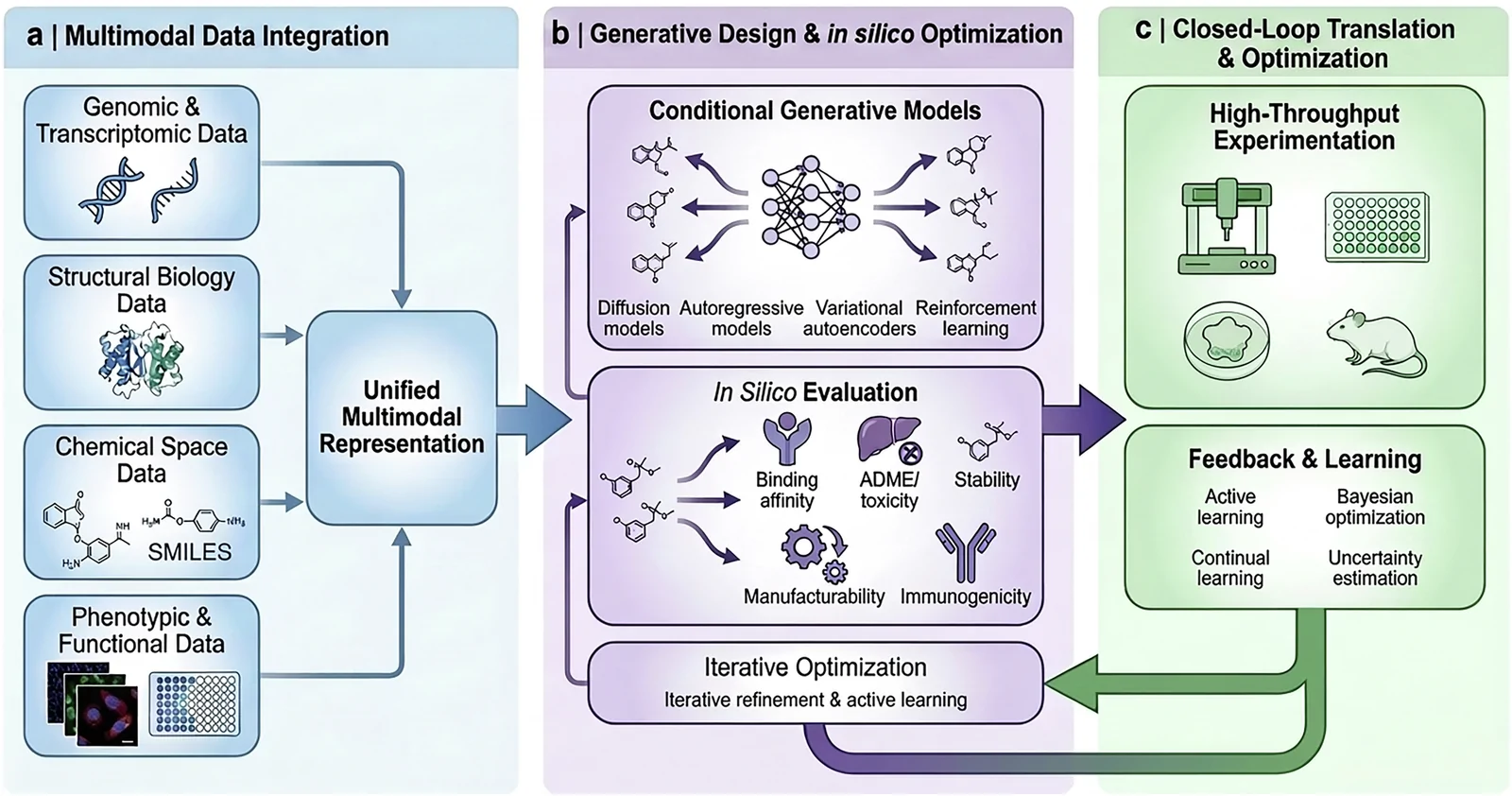
Multimodal generative AI and closed-loop optimization for de novo therapeutic design. **a** Multimodal data integration combines genomic, transcriptomic, structural, chemical, and phenotypic datasets to build unified models that learn from all these sources simultaneously. **b** Diffusion models, autoregressive architectures, variational autoencoders, and reinforcement learning-based optimization support the design of entirely new molecules and sequences. These candidates are evaluated in silico for critical properties such as binding affinity, ADME/toxicity, stability, manufacturability, and immunogenicity. **c** High-throughput experimental platforms, including automated synthesis, cell-based assays, organoid systems, and advanced functional readouts, provide feedback for active learning, Bayesian optimization, continual learning, and uncertainty-aware model refinement.

Generative models go beyond traditional rule-based methods by learning how to design molecules directly from data^65,68^ (Fig. 3a, b). Variational autoencoders (VAEs) and reinforcement learning (RL) approaches have been widely used to enable property-guided molecular optimization and de novo compound design^70,71^. VAEs encode molecules into a continuous space where similar structures are close together^72^. Property predictors associated with these embeddings enable gradient-based optimization to achieve specific safety and efficacy profiles. Generative adversarial networks (GANs) train generators to create new molecules that look like known drug-like compounds^73^. Conditional versions of these models can incorporate property constraints right into the generation process, guiding the design toward molecules with desired toxicity and pharmacokinetic properties. Reinforcement learning approaches treat molecule building as a step-by-step decision-making process, rewarding high potency and penalizing predicted toxicity^74,75^. Diffusion models, which draw on ideas from image and protein generation, gradually refine molecular graphs and allow for property-based sampling^76^. These neural network-based methods allow researchers to explore chemical space beyond the limits of established medicinal chemistry rules, though it is still important to carefully assess whether the generated compounds can be made and are relevant to the intended application. Graph-based generative models and reinforcement learning frameworks further expand the accessible chemical space for drug discovery applications^77,78^.

Instead of providing an exhaustive list of generative model classes, it is worth considering how different model classes tend to behave when applied to molecular design. For example, VAEs can ignore portions of their latent space^68^, while GANs tend to collapse to similar molecules^72^. Both of these phenomena limit the realization of chemical diversity. RL methods can become overly focused on optimizing the given metric, leading to molecules that, while high-scoring according to a predictor, fail to capture the intended medicinal-chemistry intent^73,74^. While diffusion- and flow-based methods may sample less efficiently, they can enable richer molecular diversity and greater control over structural or property-based constraints^75^. Such trade-offs can be important for constrained design problems such as fragment linker design and PROTAC generation, which require satisfying geometric constraints, ensuring synthesizability, and often multiple desired properties simultaneously. Flow-based methods like diffusion models and GFlowNets can be particularly useful in these cases, as they can realize a larger space of well-performing trade-off solutions^79^. As such, rule-based transformations can still play an important role. While not necessarily serving as replacements for generative models, they can be used as useful chemically informed priors within the TechBio 3.0 stack that encourage generated molecules to be realistic and synthesizable^66,67^.

### Integration of toxicity prediction with generative optimization

A defining feature of TechBio 3.0 is the integration of predictive and generative systems together in closed-loop optimization workflows (Fig. 3c)^80^. Rather than creating large libraries of molecules and filtering them later, today’s methods build toxicity and pharmacokinetic constraints directly into the design objectives from the very beginning. For example, in reinforcement learning, toxicity penalties are incorporated into reward functions to balance safety, potency, and Absorption, Distribution, Metabolism, and Excretion (ADME) properties from the start^81^. Multi-objective optimization strategies, such as Pareto front analysis, help manage trade-offs among different goals^82^. In VAEs, gradients in the latent space, guided by toxicity predictors, steer the search away from unsafe areas of chemical space. Generative flow networks (GFlowNets)^79^ sample a wide range of candidate molecules using composite reward functions, thereby generating diverse sets of options that cover a spectrum of viable trade-offs^82,83^. By connecting prediction and generation at the objective level, these approaches reduce the need for post hoc filtering and guide molecular design toward safer, more promising regions of chemical space. This integration moves generative chemistry away from broad exploration and toward targeted, constraint-aware molecular engineering.

Generative models often produce molecules that are structurally dissimilar to those in the property predictor’s training set. Therefore, uncertainty and error estimates must be incorporated into the design loop upstream. This requires three safeguards. Firstly, predictors must be deployed within their applicability domain so that molecules that fall outside it are flagged/penalized before being forwarded for synthesis. Secondly, uncertainty must flow through the entire workflow, from property prediction to molecular generation and final selection. Methods like deep ensembles, Bayesian inference, or conformal prediction should be used instead of single-point estimates. Lastly, active-learning experiments should use acquisition functions that provide the greatest model improvement, such as expected improvement, upper-confidence-bound sampling, or uncertainty-diversity acquisition functions. Taking these steps will close an important gap in de novo design by enabling not only the generation of novel chemistry but also the recognition of when our ADMET models lose calibration on novel scaffolds and the recovery from these situations through active experimentation^84,85^.

## Closed-loop discovery workflows

### From sequential to integrated pipelines

Traditional drug discovery followed a step-by-step approach, in which computational prediction, medicinal chemistry design, synthesis, and experimental testing were treated as separate stages^86^. TechBio 3.0 changes this by bringing all these elements together into integrated workflows. In this new model, computational predictions help scientists decide which compounds to make next, while experimental results are fed back in real time to improve the underlying models. In these closed-loop systems, different types of data are used to spot potential issues in lead compounds, AI tools propose targeted changes, and new experimental data continuously update the models through active learning^87^. Scientists choose compounds for synthesis based on predicted improvements, their feasibility to synthesize, and the new information they can provide. Each new measurement helps recalibrate the models and reduce uncertainty in important areas of chemical space^88^. Despite these advances, practical limitations remain in translating AI-driven approaches into real-world discovery pipelines at scale^89^.

### Automation and high-throughput integration

Automated synthesis platforms enable the rapid production of compounds prioritized by computational models, creating a tighter, more efficient feedback loop between prediction and experimental validation^90^. Robotic workstations perform standardized chemical reactions on proposed molecules with minimal human input. Automated purification and analytical methods ensure that the compounds being tested are exactly what was intended, while laboratory information management systems track each compound throughout the workflow, from computational design through synthesis, purification, and testing. Microfluidic and miniaturized assay platforms help reduce the amount of material needed and boost throughput for experimental toxicity testing^90^. Organ-on-a-chip systems and advanced in vitro platforms enable toxicity assessment in physiologically relevant contexts, improving the translational relevance of preclinical safety evaluation^19,21^. High-dimensional phenotypic and transcriptomic profiling approaches capture detailed cellular responses, providing rich data for training and validating multimodal predictive models^35–37^. Recursion Pharmaceuticals, for example, has built a platform that combines large-scale cellular imaging with AI-driven analysis to capture how different compounds affect cell structure and function across many cell types and conditions^17^. Their system generates large amounts of imaging data, which are analyzed by CNNs to identify promising compounds while avoiding those with toxic effects. The company’s merger with Exscientia further strengthens this approach by integrating automated synthetic chemistry, enabling a fully connected process from target identification through lead optimization.

### Human-AI collaboration in drug design

Despite advances in automation, successful drug discovery still relies on close collaboration between AI systems and human experts, who provide critical biological insights, strategic judgment, and accountability for decisions impacting patient safety^10,12,86^. Interactive visualization and explainability tools enable medicinal chemists to interrogate AI-generated compound suggestions, understand underlying model reasoning, and iteratively refine designs^58–60^. SAR frameworks and medicinal chemistry principles help contextualize how structural modifications influence molecular properties and guide optimization strategies^66,67^. Molecular similarity and chemical space analyses further support comparisons with known compounds and safety-relevant analogs^81,90^, while advances in synthesis planning enable quick assessment of the synthetic feasibility for proposed candidates^80,90^. Importantly, these systems increasingly incorporate human-in-the-loop feedback mechanisms, ensuring that AI-generated designs remain grounded in practical constraints and domain expertise while enabling iterative model improvement^74,75,91^.

## Clinical and commercial validation

### Insilico Medicine and the Rentosertib milestone

The development of Rentosertib by Insilico Medicine stands out as one of the first clinical-stage validations of AI-integrated drug discovery outputs^17^. Using its Pharma.AI platform, the company combined target identification with generative biology to pinpoint TNIK as a novel IPF target and, with generative chemistry, to design optimized inhibitors. This approach led to a timeline of approximately 18 months from target identification to preclinical candidate nomination, substantially faster than the typical industry standard of 3–5 years^16^. In a Phase IIa trial involving 71 patients with IPF, those receiving the highest dose of Rentosertib experienced a mean improvement in forced vital capacity of 98.4 mL, exceeding the minimal clinically important difference threshold of approximately 80 mL^92^. The safety profile was favorable, with no treatment-related serious adverse events reported in the highest-dose group. These results, published in Nature Medicine, represent one of the first randomized controlled trials to demonstrate positive Phase II outcomes for an AI-designed therapeutic. Beyond clinical validation, the Rentosertib program illustrates the end-to-end integration of AI across the discovery pipeline, with knowledge graph-driven target identification linking TNIK to fibrotic signaling pathways and generative models optimizing compounds for pharmacokinetic properties consistent with once-daily oral dosing^17^. Parallel advances include the clinical advancement of additional generative AI-designed small molecules and the development of AlphaFold3-based protein–ligand complex prediction frameworks, which enable structure-guided molecular design through near-experimental-accuracy modeling of binding interactions^93,94^.

As of mid-2026, no AI-designed small molecule has received FDA marketing approval. The most advanced candidate to date is Rentosertib (previously ISM001-055)^93^. The molecule has received FDA orphan drug and breakthrough therapy designations in China, and its phase IIa results were published in Nature Medicine^17^. A phase III program is expected in the back half of 2026. Rentosertib therefore represents the most definitive clinical proof point of the TechBio thesis to date, though ultimate regulatory approval remains the outstanding hurdle (Tables 5 and 6).

**Table 5:** Illustrative differences between generative paradigms

Generative paradigm	Core principle	Strengths	Limitations/Failure modes	Synthesis ease	Example tasks
Rule-based/bioisostere^66,67^	Knowledge-encoded transformations	Synthesizable; interpretable	Limited novelty; bounded by known rules	High	Lead optimization, tox-driven edits^22^
VAE^68,70^	Continuous latent space + decoder	Smooth optimization; gradient-guided	Blurry/invalid samples; posterior collapse	Moderate	Property-guided de novo design
GAN^72^	Adversarial generator/discriminator	Sharp, drug-like samples	Mode collapse; unstable training	Moderate	Focused library generation^76^
RL^73,74,78^	Reward-driven sequential build	Direct multi-objective optimization	Reward hacking; mode collapse; instability	Variable	Potency/tox trade-off, REINVENT
Diffusion^75^	Iterative denoising of graphs/3D	High diversity; strong constraint satisfaction; 3D-aware	Slow sampling; validity post-checks needed	Moderate-High	3D/linker design, pose-aware design
GFlowNet^79^	Sample proportional to reward	Diverse Pareto-spanning candidates	Reward design sensitivity; scaling	Variable	Diverse hit generation, PROTAC/linker design

Compared along several dimensions, including underlying principles, strengths, failure modes, synthetic feasibility, and example design tasks. Diffusion models and GFlowNets address limitations of existing VAE/RL approaches by satisfying constraints while maintaining molecular diversity. Example use cases for both methods include linker design and PROTAC (proteolysis- target chimera) design.

**Table 6:** Comparison of traditional sequential discovery pipelines with the closed-loop integrated workflow that defines TechBio 3.0^80–83^

Dimension	Traditional sequential pipeline	Closed-loop integrated pipeline (TechBio 3.0)
Workflow structure	Linear, stage-by-stage hand-offs	Iterative design-make-test-analyze loop
Prediction-experiment coupling	Decoupled, batch-wise	Tightly coupled, continuous feedback
Iteration cycle time	Months per cycle	Days to weeks (automated)
Model updating	Static; retrained infrequently	Online/active-learning updates from new assay data^81^
Compound prioritization	Large libraries, then filter	Acquisition-function-guided selection^81^
Synthesis and assays	Manual, low throughput	Robotic synthesis + high-throughput assays^82^
Representative examples	Conventional medicinal-chemistry campaigns	Insilico, Iambic closed-loop platforms^17,80^

### Isomorphic Labs and AlphaFold-enabled drug design

Isomorphic Labs, a spin-out from DeepMind, takes a complementary approach to drug discovery by leveraging AlphaFold technology to enable structure-based design grounded in accurate protein structure prediction^95^. The platform capitalizes on AlphaFold’s ability to predict 3D protein structures with near-experimental accuracy, a capability that is valuable for targets lacking experimentally resolved conformations^95^. The introduction of AlphaFold3 in 2024 extended these capabilities to protein–ligand complexes, enabling direct prediction of small-molecule binding interactions and expanding the scope of structure-guided drug design^56,96^. This advance is especially important for targeting specific conformational states and allosteric binding sites that are difficult to resolve experimentally. By integrating these structural predictions with generative chemistry approaches, Isomorphic Labs enables the design of molecules optimized for predicted binding poses, effectively coupling protein structure prediction with de novo molecular generation within a unified design loop^97^.

One actionable example of the TechBio 3.0 thesis is Iambic Therapeutics. Iambic is a clinical-stage, AI-native small molecule company with a platform that tightly integrates prediction, structure-based design, and automated experimentation. At its core, the company’s stack features Enchant, a multimodal model trained to predict clinical endpoints and preclinical endpoints, along with NeuralPLexer^57^, a physics-informed model for predicting protein–ligand complexes. Together, these computational assets power automated design-make-test workflows that operate on a weekly cadence. Already, Iambic’s lead asset, IAM13 63, has demonstrated early clinical activity in Phase 1/1b trials as a selective HER2 (human epidermal growth factor receptor 2) inhibitor in patients whose tumors express altered HER2^98^. To provide further context for this example, Table 7 compares prominent industrial platforms across explicit examples of TechBio 3.0 criteria.

**Table 7:** Comparative mapping of prominent industrial platforms against TechBio 3.0 criteria

Company	Multimodal representation	Generative design	Closed-loop/automation	Clinical-stage asset
Insilico Medicine (Pharma.AI)^16,17^	Yes (omics + chemistry)	Yes (Chemistry42)	Yes	Rentosertib (Ph III planned)^93^
Isomorphic Labs^56,95^	Yes (AlphaFold-based)	Yes (structure-based)	Partial	Undisclosed pipeline
Iambic Therapeutics^57^	Yes (Enchant + NeuralPLexer co-folding)	Yes (physics-informed)	Yes (weekly DMT cycles)	IAM1363 (Ph 1b)^98^

## Challenges and future directions

### Data access and sharing limitations

The effectiveness of AI models is closely tied to the quality and quantity of training data; however, pharmaceutical toxicity data are dispersed among companies, institutions, and regulatory databases, with significant barriers to sharing. Proprietary compound libraries are viewed as valuable assets, and companies are often unwilling to share them, even for compounds that have failed. While regulatory submissions contain detailed toxicity data, these data are typically not publicly accessible. Academic datasets frequently focus on screening libraries rather than the diverse, drug-like molecules most relevant to pharmaceutical research. Federated learning enables training models across distributed datasets without centralizing sensitive compound structures or assay results^99^. In this approach, participating organizations train models locally on proprietary data and share only model updates, preserving confidentiality. Organizations such as the Pistoia Alliance promote precompetitive collaboration, data standardization, and tool development, but regulatory acceptance depends on demonstrable performance under standardized, shared benchmarking frameworks^100^. Efforts to improve data interoperability increasingly emphasize adherence to FAIR (Findable, Accessible, Interoperable, Reusable) data principles to enhance accessibility and reproducibility^101^.

Beyond federated learning and precompetitive collaboration, there exist three additional means of addressing the data bottleneck. Synthetic data approaches, such as generative augmentation strategies or physics- or simulation-derived labels, can enable low-resource tasks where experimentally derived data is limited due to rare targets or poorly sampled ADMET endpoints^102^. Privacy-preserving computation allows for cross-company learning when model collaborators desire to protect intellectual property such as proprietary models or assay results during training. Secure multi-party computation, homomorphic encryption, and differential privacy each provide orthogonal methods to federated learning, and can be deployed simultaneously instead of exclusively^101^. Multi-task learning across chemical property prediction was recently demonstrated at scale by MELLODDY (Machine Learning Ledger Orchestration for Drug Discovery), which federated over 40,000 assays containing 21 million compounds from 10 pharma companies^103^. FAIR (findable, accessible, interoperable, and reproducible) data-sharing guidelines are supported by resources such as OpenBind, OpenADMET^104^, and OpenFold^105^, further strengthening reproducibility, interoperability, and open-effort contribution central to TechBio 3.0.

### Regulatory framework evolution

Regulatory agencies are increasingly developing frameworks to evaluate the use of AI in drug discovery and development. The U.S. FDA has issued discussion papers and is advancing guidance on the application of AI across the drug lifecycle, signaling a shift toward more formalized evaluation pathways for AI-enabled systems^106^. In parallel, European regulatory bodies have initiated dedicated efforts to assess the role of AI in biomedical research and regulatory science, emphasizing transparency, robustness, and alignment with established standards for safety and efficacy^100^. A central priority across these frameworks is interpretability, reflecting the need for models to provide mechanistic or biologically grounded explanations for their predictions. Regulatory agencies increasingly favor approaches that can link outputs to established biological processes, rather than relying solely on high-performing but opaque models. This shift underscores the requirement that drug development decisions be scientifically justified and auditable, particularly in high-stakes contexts such as safety assessment. As a result, there is growing interest in modeling strategies that balance predictive accuracy with explanatory insight. Collectively, these developments suggest that interpretability is becoming a foundational requirement for regulatory acceptance of AI-driven systems, rather than a secondary consideration^58,59^. The FDA’s January 2025 draft guidance, *Considerations for the Use of Artificial Intelligence to Support Regulatory Decision-Making for Drug and Biological Products*, noticed in the Federal Register as document 2024-31542, sets out a risk-based credibility-assessment framework for AI models used to support regulatory decisions across drug development, post-marketing, and manufacturing contexts^61^. The agency has also stated that this context-of-use approach is consistent with how FDA staff are already reviewing submissions that include AI components. In Europe, the EMA reflection paper on AI in the medicinal-product lifecycle, released for consultation in 2023 and finalized in 2024, provides the corresponding regulatory framework^62^. Together, these instruments, rather than broad statements about AI adoption, define the practical regulatory expectations that TechBio 3.0 systems must meet.

Regulatory expectations are also expanding beyond predictive performance to include validation under real-world conditions. Increasing emphasis is placed on uncertainty quantification, domain applicability, and robustness to distributional shift, particularly as models are applied to novel chemical space that differs from their training data^58,59^. This is especially relevant in drug discovery, where extrapolation beyond known datasets is often necessary for identifying new therapeutic candidates. At the same time, there is growing recognition of the dynamic nature of AI systems, especially in closed-loop discovery settings where models are continuously updated with new experimental data. Unlike traditional static models, these systems evolve over time, introducing challenges related to reproducibility, version control, and output consistency. Regulatory perspectives are therefore beginning to incorporate lifecycle considerations, including structured update protocols and ongoing performance monitoring. Ensuring that model behavior remains stable and reliable as underlying data distributions shift is becoming a key requirement for deployment. These trends reflect a broader shift from static model validation toward lifecycle-based regulation of AI systems, which will be critical as AI becomes more deeply integrated into experimental and clinical decision-making workflows.

### Prospective validation and clinical translation

A central limitation of current AI-driven discovery systems is that many reported gains are demonstrated on retrospective or narrowly scoped datasets, raising concerns about generalization to novel chemical space and real-world clinical outcomes. Retrospective validation is a useful step in method development, but it does not tell us much about how well these methods will work in real-world, forward-looking scenarios. These datasets often include compounds that are not representative of new chemical space, making it difficult to generalize results to truly novel molecules. For true prospective validation, we need to actually make and test compounds that AI systems have designed or prioritized, and then see whether the predictions hold up for molecules not part of the original training data. This kind of testing is expensive and takes time, especially when it involves animal studies or long-term follow-up. Ultimately, the real test is clinical outcomes. The Rentosertib results are encouraging, but we need to see more clinical data from other programs before we can draw strong conclusions about the benefits of AI in drug discovery. At present, multiple AI-designed drug candidates are advancing through preclinical and early clinical development, and their outcomes over the coming years will be critical for evaluating the practical, real-world impact of these technologies^10,12,16,17,107,108^.

### Emerging technical frontiers

Several new technical advances are set to shape the future of TechBio. Foundation models trained on billions of molecules via self-supervised learning can create general-purpose molecular representations useful for many tasks. Researchers are developing language models that read SMILES strings, GNNs trained with contrastive methods, and multimodal systems that blend structural and bioactivity data. By combining molecular design with protein structure prediction, scientists can now optimize both drugs and their targets simultaneously. Instead of relying on fixed protein structures, new approaches that model protein flexibility alongside ligand binding promise to reveal opportunities beyond those offered by traditional structure-based designs. Tools like AlphaFold3, which predict protein–ligand complexes, are making these integrated strategies possible. Quantum chemistry provides first-principles predictions of molecular properties that complement data-driven statistical models^18,29^. Hybrid approaches that integrate quantum mechanical principles with neural networks aim to achieve high predictive accuracy while maintaining computational efficiency^33,109^. The integration of physics-based and data-driven methods offers a promising path toward improving prediction reliability, particularly for novel molecules that lie outside the distribution of existing training datasets^12,15^.

Unified protein–ligand co-folding models constitute one example of a large model of critical importance to the next iteration of TechBio 3.0, alongside large language models and other biologically focused foundation models. Models like AlphaFold3^56^, Boltz, Chai, and NeuralPLexer^57^ bring protein-side structural information directly into multimodal fusion, including binding-pocket geometry and many of the affinity and selectivity determinants that may be underutilized by molecule-only representations. However, it will be important not to treat their outputs as ground truth. Known weaknesses, including uncertainty about pocket conformations, sensitivity to the depth of multiple-sequence alignment, and miscalibration for unseen protein complexes^110,111^, should be propagated through the workflow as uncertainty estimates. LLMs and other foundation models should similarly be leveraged as either broad representation extractors that capitalize on scale and self-supervised pretraining to encode biological and chemical structure inaccessible to smaller task-specific encoders^112,113^ or hypothesis generators that propose candidate compounds de novo through in-context learning, supervised fine-tuning, or reinforcement learning strategies^114,115^. Incorporating these models into the encoding and data-merging layer of the TechBio stack, and AI-assisted review into the compliance layer, is therefore a major focus for TechBio 3.0 development.

### Future challenges and opportunities

Several multimodal modeling challenges must be addressed before automated synthesis is commonly used in drug discovery. Missing modalities: Because transcriptomic, structural, and assay data are unavailable or noisy for most compounds, models must be robust to modality dropout, noisy annotations, or missing values altogether^54^. Misaligned data distributions: Cross-modal alignment is challenging when data from different sources are measured under different experimental conditions; making chemical structures, protein complexes, omics profiles, and assay readings compatible will require careful attention^54^. Evaluation of signal contribution by modality: A multimodal model can be effectively treated as a unimodal model if it ignores all but one modality. Tools like ablation studies and Shapley-style feature attribution can help reveal when a model fails to meaningfully integrate each of its inputs^59^. Scale: As the number of parameters and integrated modalities increases, scaling requirements will become a bottleneck for training, deployment, and interpretation.

The experimental bottleneck matters just as much. After all, an AI-designed molecule is worthless if it can’t be made. As such, synthetic accessibility, including retrosynthetic feasibility and route cost, should be assessed during design rather than after candidate selection^90^. Automated synthesis planning is far from perfect: it introduces prediction errors and reaction-yield uncertainty, which can accumulate across an entire proposed route^90^, and robotics introduce**s** additional errors during execution^82^. High-throughput experimentation can speed up the loop, but doesn’t magically overcome limits of assay capacity, cycle time, and reproducibility. To truly close the loop, robotic platforms, acquisition functions, and model retraining will all need to work together as part of a single calibrated system, rather than disjointed steps^81^. Jointly tackling these modeling and experimental challenges, rather than independently optimizing algorithms and laboratories, is the core engineering hurdle for TechBio 3.0.

## Conclusion

TechBio 3.0 marks a structural evolution in pharmaceutical drug discovery, shifting from experimental AI applications to an integrated infrastructure spanning development workflows. Multimodal approaches have outperformed unimodal methods in reported benchmark comparisons by better capturing biological complexity^30,31^. The combination of predictive models with generative chemistry enables closed-loop systems that can both identify problematic compounds and design improved alternatives. Hierarchical attention mechanisms provide the interpretability needed for regulatory acceptance and informed scientific decision-making. Ultimately, clinical outcomes will determine whether AI-driven discovery systems lead to better success rates, shorter development timelines, and improved safety. While the technological infrastructure is rapidly emerging, the lasting impact will depend on thorough validation and reproducible clinical results. In the coming decade, the convergence of multimodal modeling, generative design, and automated experimentation could redefine drug discovery as a data-driven engineering discipline, shifting the field from empirical optimization toward predictive and controllable therapeutic design.

## Data Availability

No datasets were generated or analyzed during the current study.
